# Supplementary material for: Down-Regulation of Neogenin Decreases Proliferation and Differentiation of Spermatogonia during the Early Phase of Spermatogenesis
Source: Int J Mol Sci. 2022 Nov 25;23(23):14761. doi: 10.3390/ijms232314761 (PMC9738271; doi:10.3390/ijms232314761)
Supplement: Supplementary file 1 [file ijms-23-14761-s001.zip › ijms-1874194-supplementary.pdf]

Table S1. Primer sequence for RT-PCR.

Gene		Primer sequences (5'-3')	
Neogenin	Neogenin	F	GTA TGT CGC CTC GCT ACC TG
		R	GCC ACA GAG AAG TCA TCG GA
Oct3/4	Octamer-binding transcription factor 4	F	CAC GAG TGG AAA GCA ACT CA
		R	AGA TGG TGG TCT GGC TGA AC
Sox2	Sox2	F	TAGAGCTAGACTCCGGGCGATGA
		R	TTGCCTTAAACAAGACCACGAAA
Nanog	Nanog	F	CAC CCA CCC ATG CTA GTC TT
		R	ACC CTC AAA CTC CTG GTC CT
β-Actin	β-Actin	F	CAT TGC TGA CAG GAT GCA GAA GG
		R	TGC TGG AAG GTG GAC AGT GAG G

Table S2. sgRNA (synthetic guide RNA) sequence for CRISPR/Cas9 neogenin knock-out study.

ID	sgRNA sequence	Target	Transcript ID	Distance to TSS	Chromosome	Location
**1**	ACATTCCGGCCGAGAGCCGG	Neo1	NM_001042752	+99	9	59036342
**3**	CGCCCGGAAGTCTTTCCCCT	Neo1	NM_001042752	+165	9	59036276
**4**	CTACCGTGAGGAGCCCGAGT	Neo1	NM_001042752	+32	9	59036409
